# Naltrexone and the Treatment of Alcohol Dependence

**Published:** 1994

**Authors:** Joseph R. Volpicelli, Karen L. Clay, Nathan T. Watson, Laura A. Volpicelli

**Affiliations:** Joseph R. Volpicelli, M.D., Ph.D., is an assistant professor at the University of Pennsylvania Medical Center, Philadelphia, Pennsylvania. Karen L. Clay, B.A., Nathan T. Watson, B.A., and Laura A. Volpicelli, B.A., are research assistants at the University of Pennsylvania Medical Center, Philadelphia, Pennsylvania

## Abstract

There currently is a great demand for effective medications to reduce the high relapse rates that occur in the early stages of treatment for alcohol dependence. Recent clinical trials of the opiate antagonist naltrexone have shown that this medication significantly decreases excessive alcohol drinking.

Although current treatments for alcohol dependence^1^ can safely and effectively help patients stop drinking alcohol, they are only modestly successful in reducing patients’ tendencies to relapse, or return to excessive alcohol consumption. About one-half of all patients who complete an inpatient or residential psychosocial treatment program relapse within the first 3 months of treatment, causing this period of recovery to be critical for maintaining abstinence (Miller and Hester 1986; Nathan 1986). Because of the high relapse rates that occur early in recovery, considerable interest has developed in finding medications that help prevent a quick return to alcohol abuse. A longer period of abstinence is considered to increase eventual treatment success. The most commonly used and researched pharmacological agents include disulfiram; lithium; and those that increase activity of the serotonin system (i.e., the system that affects mood states), such as fluoxetine (Prozac^®^) (see the article by Anton, pp. 265–271). However, none of these compounds have been shown in studies to work better than placebo in a clinical population.

A promising new approach to the problem of relapse is the use of opiate antagonists. These substances block specific portions of neurons in the brain—opiate receptors—that are stimulated by opioids,^2^ such as morphine. Opiate receptors also are targets for alcohol’s activity in the brain. Blocking the receptors seems to prevent many of the positive consequences of drinking alcohol, such as the alcohol “high,” thereby decreasing the likelihood of excessive alcohol consumption.

One opiate antagonist, naltrexone, appears to be a safe and effective adjunct for the early treatment of alcohol dependence. This article reviews research suggesting that opiate antagonists reduce excessive alcohol consumption in animal models and presents supporting data from two clinical samples of alcohol-dependent patients given the drug naltrexone. Finally, the article describes how naltrexone may work to reduce excessive drinking.

## Experimental Background

**Figure 1:**
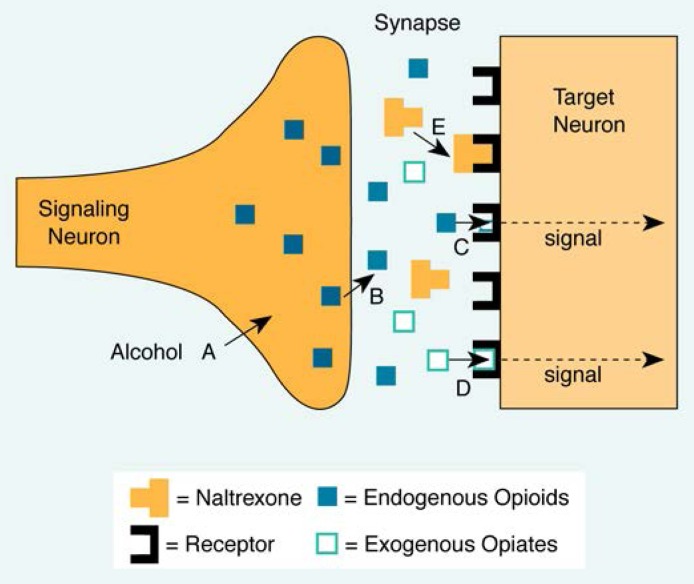
Opiate receptor activation of a nerve and blockage of the signal by naltrexone. A) Alcohol is thought to stimulate the release of endogenous opioids, which may produce the euphoric feelings associated with alcohol consumption. B) Endogenous opioids (e.g., beta-endorphin) are released into the synapse (the space between the signaling and target neurons) and C) stimulate activity at opiate receptors, which produces a signal in the target neuron. D) Exogenous opiates such as morphine also stimulate opiate receptors. E) Naltrexone is thought to block opioids from activating opiate receptors.

For one-quarter of a century, researchers have shown that alcohol consumption can alter the activity of opiate receptors (Cohen and Collins 1970; Davis and Walsh 1970). These receptors exist in the brain and normally are stimulated by naturally occurring substances (endogenous opioids) that are released by neurons in response to physical and emotional pain. Drugs, such as morphine, that mimic the effects of endogenous opioids also may stimulate opiate receptors. Other drugs, such as alcohol, may enhance opiate receptor activity indirectly, perhaps by stimulating the release of endogenous opioids. In support of this theory, animal studies have demonstrated important interactions between alcohol consumption and increases in opiate receptor activity. Specifically, alcohol consumption stimulates opiate receptor activity; is influenced by the use of opiates, such as morphine; and is reduced by opiate antagonists, such as naltrexone (figure 1). Each of these findings is described below.

### Alcohol’s Effects on Opiate Receptor Activity

Several mechanisms by which alcohol may indirectly affect opiate receptor activity have been suggested, but the one presented here appears most important in understanding alcohol dependence. Accumulating evidence, including studies in humans, shows that alcohol stimulates the release of endogenous opioids, such as beta-endorphin, normally used by the body to mitigate pain, and other endorphins that produce pleasurable effects (see reviews by Gianoulakis 1993; Volpicelli et al. 1994).

This release of endorphins may explain why some strains of rats and some people drink excessive amounts of alcohol. For example, rats bred to have a high preference for alcohol show enhanced beta-endorphin release when they consume alcohol compared with rats bred for a low preference for alcohol (deWaele et al. 1992). Similarly, in a study conducted by Gianoulakis and colleagues (1990), male social drinkers who were at increased risk for alcohol abuse, because they had alcohol-dependent parents, experienced a 170-percent increase in peripheral beta-endorphin levels after consuming a moderate dose of alcohol. In contrast, male social drinkers without alcohol-dependent parents did not show any increase in beta-endorphin levels after drinking the same amount of alcohol. Thus, enhanced release of endorphins induced even by small amounts of alcohol may increase the risk for alcohol abuse, and the more a person drinks, the more endorphins appear to be released.

### Opiate Effects on Alcohol Consumption

Many studies show that the administration of opiates affects alcohol consumption (see table 1). For example, rats given a single 30 milligrams per kilogram (mg/kg) morphine injection (a high dose) decreased their alcohol but not their water consumption, demonstrating that morphine’s effect was restricted to alcohol (Sinclair 1974). Ho and colleagues (1976) also found that rats decreased their alcohol consumption in inverse proportion to their morphine dose. In this study, rats given an incremental dose regimen (i.e., 10, 30, 60 mg/kg) of morphine showed a corresponding decrease in alcohol consumption.

**Figure 2:**
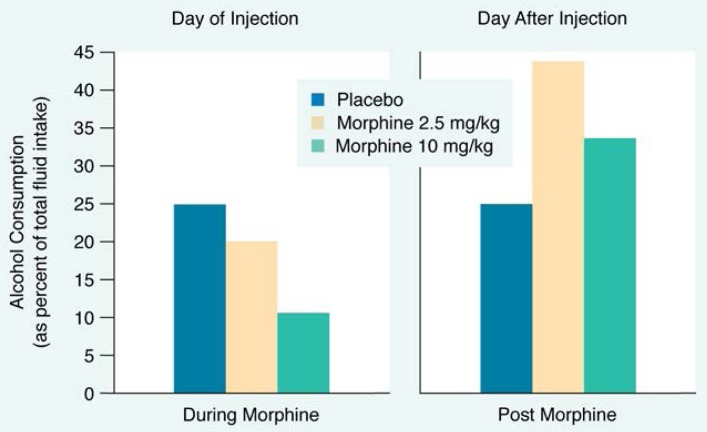
The effects of injection with morphine on alcohol consumption are shown. While under the effects of low (2.5 milligrams per kilogram [mg/kg]) or high (10 mg/kg) doses of morphine, rats decreased their alcohol consumption in inverse proportion to the dose of morphine they had received. The day after the morphine injections, however, both sets of rats increased their alcohol consumption. Rats that had been given low doses of morphine greatly increased their alcohol consumption and those given high doses increased their alcohol consumption to a lesser degree SOURCE: Volpicelli et al. 1991.

Although opiate use can reduce preference for alcohol, preference dramatically increases during opiate withdrawal. For example, Volpicelli and colleagues (1991) found that rats with free access to both alcohol and water decreased their alcohol consumption, compared with control rats, when injected with morphine. The day after receiving the injections, however, the morphine-injected rats drank approximately twice as much alcohol as the control rats (figure 2). These results further support an inverse relationship between opiate receptor activity and alcohol consumption. An increase in activity at opiate receptors causes a decrease in alcohol consumption, whereas subsequent opiate withdrawal causes an increase in alcohol consumption.

An important exception to this relationship comes from the research conducted by Reid and associates (1991), in which small doses of morphine were found to increase alcohol drinking transiently in rats given limited access to alcohol or water. For example, rats with 2-hour access to alcohol or water that received a low dose of morphine (generally less than 2.5 mg/kg) typically drank more of an alcohol solution than did rats injected with saline.

The effect of small doses of opioids to increase alcohol drinking is similar to receiving an appetizer before dinner. A small amount of a pleasurable substance can increase the motivation to consume more of that substance. This appetizer, or priming, effect has been observed across a variety of addictive drugs. For example, rats that have stopped signaling (by pressing a bar) to obtain cocaine will respond again after receiving a small dose of cocaine (Stewart 1983). Similarly, human cocaine-dependent patients often report that sampling cocaine enhances the motivation to use more cocaine, creating a vicious addictive cycle (Jaffe et al. 1989).

A similar addictive cycle may occur in animals or humans who drink alcohol excessively. Like the injection of a small dose of morphine, alcohol consumption may lead to modest increases in opiate receptor activity. This heightened opiate receptor activity primes, or enhances, the motivation to drink more alcohol. Thus a vicious cycle could be established for people at risk for developing alcohol abuse. These people (such as the high-risk subjects mentioned above in the study by Gianoulakis and colleagues [1990]) may find that one drink increases the motivation and craving for the next drink, and this may explain why some alcohol-dependent people find it difficult to control their alcohol consumption once they have begun to drink. This mechanism also suggests that the use of an opiate receptor blocker, such as naltrexone, could break the vicious cycle and thus reduce excessive alcohol consumption. (Table 1 summarizes studies that further demonstrate the contradictory effects of opioids on alcohol consumption in animals.)

### Opiate Antagonist Effects on Alcohol Consumption

An extensive body of literature shows that compounds blocking opiate receptors—opiate antagonists—reduce alcohol drinking in animals (table 1). Many of these studies have been performed using naloxone, a short-acting, injected opiate antagonist. For example, Marfaing-Jallat and colleagues (1983) found that naloxone reduces alcohol preference in rats given a choice between water and alcohol. Numerous researchers have replicated these results in a variety of studies (e.g., Samson and Doyle 1985; Froehlich et al. 1990).

Several animal studies also have used the opiate antagonist naltrexone to reduce alcohol consumption. Naltrexone is a longer acting compound developed from naloxone that is used as a treatment for opiate addiction. For example, Altshuler and colleagues (1980) found that naltrexone decreased alcohol intake among rhesus monkeys trained to press levers to obtain doses of intravenous alcohol. Similarly, in a rat model of stress-induced excessive alcohol consumption, researchers found that naltrexone injections completely blocked the animals’ stress-induced increases in alcohol consumption (Volpicelli et al. 1986).

## Naltrexone Studies in Alcohol-Dependent People

The preclinical research studies discussed above have served as the impetus to test naltrexone in the treatment of alcohol-dependent patients. Overall, animal studies suggest that alcohol produces important pharmacological effects that enhance opiate receptor activity, that opiate receptor activity can influence the desire to drink alcohol, and that opiate antagonists can block alcohol’s rewarding effects.

### The Volpicelli Study

To determine naltrexone’s efficacy in reducing rates of relapse in alcohol-dependent people, Volpicelli and colleagues (1992) performed a 12-week, double-blind clinical trial in which neither the investigators nor the subjects knew if naltrexone or placebo was being administered.^3^ The researchers administered either 50 mg of naltrexone or placebo daily on an outpatient basis to 70 alcohol-dependent male veterans, predominantly African-American and unemployed, who had been drinking heavily for an average of 20 years. In addition to their medication, all subjects received psychosocial therapy in the hospital that included supportive alcoholism counseling, relapse prevention therapy, and referral to Alcoholics Anonymous meetings. Primary outcome measures included self-reports of alcohol drinking and craving (i.e., the desire to drink as assessed on a 10-point scale) and liver damage as determined by increased liver enzyme levels. Alcohol relapse also was assessed as a measure different from sampling alcohol, or “slipping.”

#### Craving

**Figure 3:**
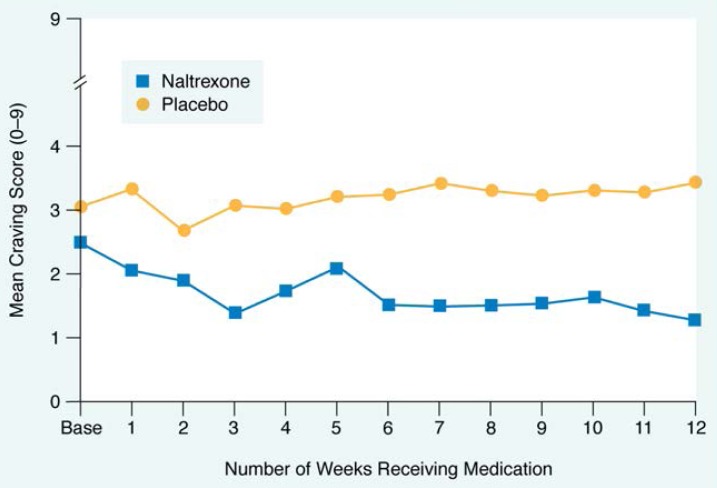
Naltrexone’s effects on alcohol craving are shown. Each point represents the average craving score for all patients in each group. Alcohol craving for patients receiving placebo did not change over the 12 weeks of the study. Alcohol craving for the group taking naltrexone decreased from its original mean score and remained at low levels while the group was on medication SOURCE: Volpicelli et al. 1992.

The naltrexone-treated subjects experienced a gradual decline in alcohol craving during the 12 weeks of the study. The placebo-treated subjects, however, had higher overall levels of craving throughout the study and experienced no reduction in craving (figure 3).

#### Effects on Drinking

Possibly as a consequence of their reduced desire to drink, naltrexone-treated subjects reported less alcohol consumption than the placebo-treated subjects. Although the percentage of naltrexone-treated patients who drank any alcohol during the study was equal to the percentage of placebo-treated patients who drank, the naltrexone-treated patients who slipped consumed alcohol on fewer days than did placebo-treated patients. Of the subjects who slipped, the placebo-treated group drank alcohol on nearly four times as many days as the naltrexone-treated subjects (14.0 percent of the study days versus 3.6 percent of the study days).

To support the results obtained from the subjects’ self-reports of drinking, excessive alcohol consumption was assessed by monitoring elevations in liver enzymes that signify liver damage. Although the naltrexone and placebo groups did not differ significantly from each other, many naltrexone subjects had lower liver enzyme values.

#### Effects on Relapse

An important measure of naltrexone’s beneficial effects can be seen when looking at the loss of control over alcohol drinking, or alcohol relapse, in these patients. For research purposes, Volpicelli and colleagues defined alcohol relapse in the subjects as follows: consuming five or more drinks on a particular drinking occasion, presenting for treatment with a blood alcohol concentration greater than 100 mg percent (legal intoxication), or consuming alcohol five or more times during the previous week.

**Figure 4:**
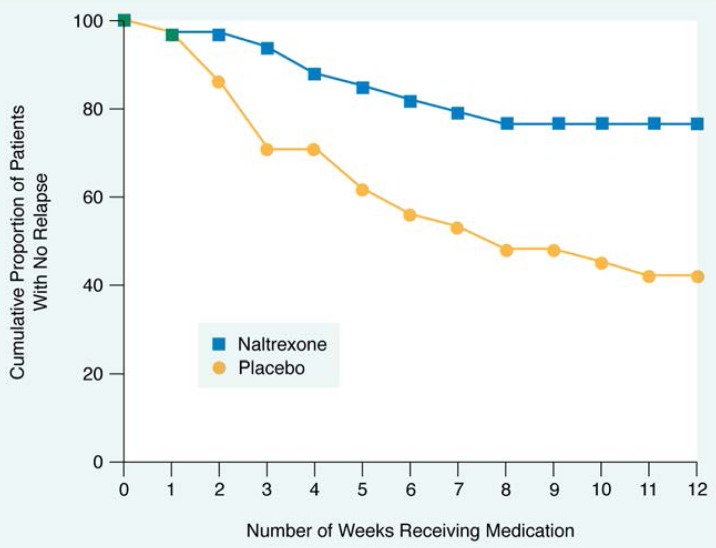
Effects of naltrexone on relapse rates among patients in treatment for alcohol dependence are shown. Only 25 percent of patients given naltrexone relapsed into alcohol abuse and dependence during the study, whereas more than 50 percent of the patients given the placebo relapsed SOURCE: Volpicelli et al. 1992.

The five-drinks-per-drinking-occasion criterion was the most sensitive measure of relapse, because virtually every subject who met the other relapse criteria also met the five-drink criterion. The five-drink criterion was based on data suggesting that alcohol binges of five or more drinks for males and four or more drinks for females are associated with biopsychosocial problems (i.e., any type of alcohol-related problems; Knupfer 1984). Volpicelli and colleagues’ clinical data (1992) further suggest that subjects who met the relapse criteria were likely to meet other criteria for alcohol dependence, such as elevated liver enzymes; a feeling of loss of control over alcohol use; interpersonal problems; and, in the subjects who worked, occupational problems. In contrast, none of the subjects who drank alcohol but who did not meet relapse criteria had any biopsychosocial problems associated with their alcohol use. Using this definition of relapse, about one-half of the placebo-treated subjects relapsed during the 12 weeks of the study, whereas fewer than one-fourth of the naltrexone-treated subjects relapsed (figure 4).

#### Side Effects

The study’s psychiatrist assessed treatment side effects noticed by the subjects every 4 weeks. Of the 70 subjects enrolled, only 2 subjects withdrew from the study because they were unable to tolerate the side effects. Both subjects were taking naltrexone and complained of nausea. This finding was not significant. Except for nausea, all other side effects were mild in nature and did not differ between groups.

### The O’Malley Study

Another trial of naltrexone in a distinct outpatient population incorporated two different psychosocial therapies and further demonstrated the medication’s effectiveness at reducing slips and preventing alcohol relapse among people in alcoholism treatment. In this study, O’Malley and colleagues (1992) administered 50 mg of naltrexone or placebo for 12 weeks to 97 subjects (72 men and 25 women), predominately white and employed full time.

Overall, O’Malley and colleagues found that naltrexone treatment reduced alcohol consumption and lowered relapse rates. In addition, the researchers investigated the interaction of naltrexone with two types of psychosocial therapy. One type of therapy, coping skills therapy, taught patients strategies to cope with alcohol craving and to identify and cope with lifestyle factors that may lead to alcohol relapse (e.g., learning to manage anger or to communicate in close relationships). The other type of therapy, supportive therapy, involved patients meeting with a nondirective therapist (i.e., one who encouraged abstinence without teaching specific coping skills) to discuss issues relating to abstinence and treatment.

Similar to the Volpicelli study (1992), O’Malley defined relapse as drinking five or more drinks on an occasion for male subjects and four or more drinks on an occasion for females. Using these definitions, O’Malley and colleagues found that naltrexone reduced overall relapse rates among subjects by approximately one-half. Also, subjects taking naltrexone reported drinking on only 4.3 percent of the study days, whereas placebo subjects consumed alcohol on 9.9 percent of the study days.

An interaction also appeared to occur between the use of medication and the type of psychotherapy group attended. Subjects taking naltrexone and receiving supportive therapy were less likely to sample a drink for the first time after beginning treatment than were subjects in the other treatment groups. Subjects taking naltrexone and receiving coping skills therapy, however, slipped as often as the placebo subjects but were less likely to relapse than any other group once a slip occurred.

The study by O’Malley and colleagues suggests that a program combining naltrexone treatment with coping strategies seems particularly effective in reducing alcohol relapse, alcohol craving, and loss of control over alcohol drinking.

## Evidence for Naltrexone’s Mechanism of Action

Both clinical trials of naltrexone demonstrated a significant reduction of alcohol relapse in those subjects taking naltrexone compared with subjects taking placebo. For example, nearly all the subjects taking placebo who slipped during the study went on to meet relapse criteria. In comparison, only one-half of the subjects taking naltrexone who slipped during the study relapsed (Volpicelli et al. 1992). Although naltrexone may not reduce the risk of slipping, it appears to stop a slip from becoming a relapse. Furthermore, naltrexone seems to prevent the loss of control over alcohol consumption and the return to alcohol abuse and dependence typical of many alcohol-dependent patients in treatment. It may do so by diminishing alcohol’s pleasurable effects.

**Figure 5:**
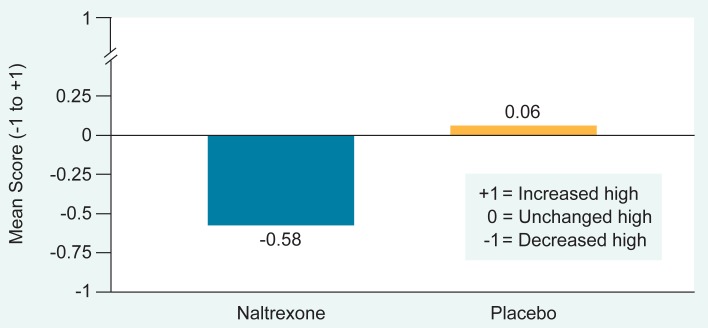
Naltrexone’s effects on the alcohol “high” felt by patients who slipped (tasted alcohol during the study) are shown. Patients compared the high they experienced at this time with the high they felt from alcohol before they entered treatment. Each bar represents the average score for all patients in each group. On a scale from negative 1 to positive 1, patients given naltrexone who slipped during the study rated their alcohol high as an average of −0.58. Patients on the placebo rated the high as an average of 0.06. SOURCE: Volpicelli et al. 1992.

In Volpicelli and colleagues’ (1992) clinical trial, subjects who experienced a slip during the trial were asked to report the subjective effects of alcohol and to rate the “high,” or euphoria, from alcohol during their slip on a three-point scale (from −1 to +1; see figure 5). The average rating was −0.58, implying that naltrexone-treated patients experienced a decrease in their high obtained from drinking alcohol. The placebo-treated patients, however, reported no change in alcohol euphoria (mean rating, +0.06) (Volpicelli et al. 1992). This suggests that the decrease in alcohol consumption in the naltrexone group was attributable to the reduction in the high normally caused by alcohol consumption. However, in this study with alcohol-dependent subjects, the amount of alcohol consumed during a slip was not controlled. Because naltrexone-treated subjects also drank less alcohol than placebo-treated subjects did during their slips, the diminished high may instead have been a consequence of their reduced alcohol intake. The studies with nondependent social drinkers reviewed below (Swift et al. in press) support naltrexone’s ability to reduce alcohol’s high.

### Why Might Naltrexone Work?

Naltrexone may reduce alcohol craving that alcohol-dependent people often feel. Alcohol dependence is characterized by a loss of control over drinking, expressed by recovering alcoholics as feeling that “1 drink is too many and 100 is not enough.” Many alcohol-dependent patients report that the more alcohol they drink, the greater their craving becomes, and they are unable to stop drinking once they begin. As discussed earlier, this vicious cycle may be initiated by alcohol-induced increases in opiate receptor activity, leading to an increased motivation to drink alcohol. Naltrexone, by blocking opiate receptors, should disrupt this cycle by obstructing the enhanced opiate receptor activity induced by alcohol consumption.

Naltrexone also may mitigate the pleasure people experience when they drink. The animal data reviewed here suggest that alcohol is rewarding in part because of its effect on opiate receptor activity. In some people, alcohol may produce a morphinelike high. If this is true, it would be likely that these people would not experience an alcohol high while taking naltrexone.

To determine if naltrexone reduces the pleasurable effects associated with drinking, studies must be conducted in which the amount of alcohol consumed is consistent across all groups. Giving alcohol to recovering alcoholics raises ethical concerns; therefore, the effects of naltrexone on reducing drinking pleasure are best studied using nondependent social drinkers. In one such study, Swift and colleagues (in press) found that after a standard dose of alcohol (e.g., a 5-ounce glass of wine), social drinkers who took naltrexone experienced less euphoria than the subjects who took the placebo.

Naltrexone not only reduced the pleasurable effects associated with drinking, but it also increased the less desirable sedative effects of alcohol relative to the effects felt by the placebo group. Thus, although the naltrexone-treated subjects experienced the negative effects of alcohol consumption, they did not experience the positive effect of euphoria that is a motivation for drinking alcohol.

Alternately, naltrexone may interact with alcohol consumption to produce an aversive reaction, similar to the presumed mechanism for disulfiram. For example, some subjects taking naltrexone reported that following alcohol consumption they felt “hungover” or nauseous a few hours after drinking. Consequently, they did not enjoy the alcohol. In social drinkers, Swift and colleagues (in press) have reported similar results: some subjects taking naltrexone and drinking alcohol vomited. Taking naltrexone alone or consuming alcohol alone was not associated with vomiting.

## Conclusions

Naltrexone has been shown in two independent, double-blind clinical trials to reduce alcohol consumption effectively in alcohol-dependent patients when used in conjunction with psychosocial therapy. Alcohol-dependent patients who take naltrexone show significantly reduced drinking levels and alcohol relapse and report a reduced high from alcohol.

It is unlikely that any single medication will be effective for all alcohol-dependent subjects. Further research is needed to identify “responder” subpopulations (whose characteristics predispose them to have the most success with a certain medication) so that the appropriate pharmacological interventions for each group can be given. Additional research on the most effective dose of naltrexone in the treatment of alcohol dependence and the most effective treatment duration also should be performed before naltrexone can be widely used in clinical settings. Even if naltrexone is found to be pharmacologically effective, a patient’s motivation to refrain from drinking must be reinforced, or it is likely that poor compliance will limit naltrexone’s clinical utility. The clinical studies reported here included subjects who were actively engaged in psychotherapy. Thus, naltrexone is best used as a part of a treatment program that includes psychosocial interventions. At this point, findings indicate that naltrexone has significant promise as a safe pharmacological adjunct in treating alcohol dependence.

## Figures and Tables

**Table 1:** Alcohol produces some of its effects by interacting with opiate receptors in the brain, which normally are activated in response to pain by naturally occurring substances called opioids. Substances that mimic opioid function, such as morphine, are studied to elucidate alcohols interaction with this system. These studies offer evidence of the contradictory effects of high and low doses of opioids on alcohol consumption in rats as well as the effects of opiate antagonists, substances that block opiate receptors, on alcohol consumption. In general, higher doses of opioids decreased alcohol consumption by the rats, whereas low doses increased consumption. Doses of opioid antagonists, in contrast, decreased alcohol consumption regardless of the dose amount.

Effects of Opioids and Opioid Antagonists on Alcohol Drinking in Animals		

Study^1^	Drug/Amount	Results^2^
**Moderate to High Doses of Opioids**		
Beaman et al. (1984)	Morphine 10 mg/kg	Decreased alcohol intake
Ho et al. (1976)	Morphine 10, 30, 60 mg/kg	Decreased alcohol consumption
Reid et al. (1987)	Morphine 7.5, 20.0 mg/kg	Decreased alcohol intake
Ross et al. (1976)	Morphine sulfate 7.5 mg/kg	Decreased alcohol intake
Sinclair et al. (1974)	Morphine 30 mg/kg	Decreased alcohol intake
Volpicelli et al. (1991)	Morphine 10.0 mg/kg	Decreased alcohol intake

**Low Doses of Opioids**		
Beaman et al. (1984)	Morphine 2.5 mg/kg	Increased alcohol intake
Chol et al. (1990)	Morphine 0.6 mg/kg	Increased alcohol intake
Czirr et al. (1987)	Fentanyl 5, 10, 20, 40 μg/kg	Dose-related increase in alcohol intake
Hubbell et al. (1986)	Morphine 2.5 mg/kg	Increased alcohol intake
Hubbell et al. (1988)	Morphine 0.01, 0.1, 0.3, 0.41, 0.56 1.0 mg/kg	Increased alcohol intake (doses > 0.41 mg/kg)
Reid et al. (1991)	Morphine 2.0 mg/kg	Increased alcohol intake

**Doses of Opiate Antagonists**		
Altshuler et al. (1980)	Naltrexone 1, 3, 5 g/kg	Decreased responding^3^ to receive alcohol
Beaman et al. (1984)	Naloxone 3 mg/kg	Decreased total fluid intake
DeWitte (1984)	Naloxone 1 mg/kg	Reduced alcohol drinking
Froehlich et al. (1991)	Naloxone 0.5–3.0 mg/kg	Reduced alcohol preference
	IC174864 0.5–3.0 mg/kg	Reduced alcohol preference
Froehlich et al. (1990)	Naloxone 0.05–18.0 mg/kg	Reduced alcohol intake
Hubbell et al. (1988)	Naloxone 3.0 mg/kg	Reduced alcohol intake
	LY117413 0.01, 0.1, 1.0, 3.0, 10.0 mg/kg	Reduced alcohol intake
Marfaing-Jallat et al. (1983)	Naloxone 1 mg/kg	Reduced alcohol consumption
Samson and Doyle (1985)	Naloxone 5, 10, 20 mg/kg	20 mg dose decreased alcohol intake; at 5–20 mg, no change
Volpicelli et al. (1986)	Naltrexone 10 mg/kg	Blocked alcohol intake

1 Full citations of studies presented here are available from the author. -

2 All terms in this column reflect how the drug given altered the animals alcohol intake. The specific terminology is -used in each case as it was in the study. -

3 Animals in this study were trained to perform a task, or to respond, to receive an intravenous dose of alcohol. -

## Footnotes

1 Terms such as “alcohol abuse” and “alcohol dependence” are defined in this article using criteria from the American Psychiatric Association’s *Diagnostic and Statistical Manual of Mental Disorders, Fourth Edition* (1994).

2 The term “opioid” is a general category of compounds encompassing opiates, derived directly from the opium plant, such as morphine and heroin, as well as other compounds that occur naturally in the body and act like opiates, such as endogenous opioids.

3 No systematic validity checks were conducted to determine if subjects could determine whether they were taking active medication or placebo. Data collected using normal volunteers, however, indicate that subjects cannot differentiate between naltrexone or placebo; there are no discriminating variables until a subject begins drinking.
